# Malaria patterns across altitudinal zones of Mount Elgon following intensified control and prevention programs in Uganda

**DOI:** 10.1186/s12879-020-05158-5

**Published:** 2020-06-17

**Authors:** Aggrey Siya, Bosco John Kalule, Benard Ssentongo, Akim Tafadzwa Lukwa, Anthony Egeru

**Affiliations:** 1 College of Veterinary Medicine, Animal Resources and Biosecurity, Makerere University, P.O. Box 7062, Kampala, Uganda; 2 Centre for Invasion Biology, Department of Botany and Zoology, Stellenbosch University, Stellenbosch, South Africa; 3 College of Agricultural and Environmental Sciences, Makerere University, P.O. Box 7062, Kampala, Uganda; 4 Faculty of Health Sciences, School of Public Health and Family Medicine, Health Economics Unit, University of Cape Town, Cape Town, South Africa

**Keywords:** Altitude, Patterns, Ecohealth, Malaria, Time series, Climate change, Infectious diseases

## Abstract

**Background:**

Malaria remains a major tropical vector-borne disease of immense public health concern owing to its debilitating effects in sub-Saharan Africa. Over the past 30 years, the high altitude areas in Eastern Africa have been reported to experience increased cases of malaria. Governments including that of the Republic of Uganda have responded through intensifying programs that can potentially minimize malaria transmission while reducing associated fatalities. However, malaria patterns following these intensified control and prevention interventions in the changing climate remains widely unexplored in East African highland regions. This study thus analyzed malaria patterns across altitudinal zones of Mount Elgon, Uganda.

**Methods:**

Times-series data on malaria cases (2011–2017) from five level III local health centers occurring across three altitudinal zones; low, mid and high altitude was utilized. Inverse Distance Weighted (IDW) interpolation regression and Mann Kendall trend test were used to analyze malaria patterns. Vegetation attributes from the three altitudinal zones were analyzed using Normalized Difference Vegetation Index (NDVI) was used to determine the Autoregressive Integrated Moving Average (ARIMA) model was used to project malaria patterns for a 7 year period.

**Results:**

Malaria across the three zones declined over the study period. The hotspots for malaria were highly variable over time in all the three zones. Rainfall played a significant role in influencing malaria burdens across the three zones. Vegetation had a significant influence on malaria in the higher altitudes. Meanwhile, in the lower altitude, human population had a significant positive correlation with malaria cases.

**Conclusions:**

Despite observed decline in malaria cases across the three altitudinal zones, the high altitude zone became a malaria hotspot as cases variably occurred in the zone. Rainfall played the biggest role in malaria trends. Human population appeared to influence malaria incidences in the low altitude areas partly due to population concentration in this zone. Malaria control interventions ought to be strengthened and strategically designed to achieve no malaria cases across all the altitudinal zones. Integration of climate information within malaria interventions can also strengthen eradication strategies of malaria in such differentiated altitudinal zones.

## Background

According to the World Health Organization (WHO), malaria cases in the year – 2018 were estimated at 228 million cases of malaria worldwide with 405,000 deaths [1]. Children under 5 years accounted for the largest (67%) deaths [1]. Of the total number of cases globally, Africa was a home to 93% of malaria cases and 94% of malaria deaths [1]. In 2013, it was estimated that a total of 437,000 African children died before their fifth birthday due to malaria and the disease caused an estimated global 453,000 under-five deaths in the same year − 2013 [2]. Through bites of infected mosquitoes, disease causing parasites are transmitted to humans [1, 3]. Transmission dynamics area shaped by the environmental conditions, lifespan of the vector and the host’s immunity [1]. Climatic conditions influence the lifespan of the vector while host’s immunity reduce the risk of malaria infection in causing malaria disease in human body [1]. Several interventions have been implemented over the last decade and have led to observed decline in the malaria burden in sub-Saharan Africa. These interventions have aimed at avoiding mosquito bites through the use of repellents or insecticide treated bed nets, and specific medicines to prevent malaria. However, it still remains a major public health threat in areas within the tropical and subtropical region [4, 5].

Malaria occurrence has traditionally been observed in the low-land areas, bogs and generally in the plains within the tropical regions [6]. Comparative analysis have shown the occurrence of such patterns in Africa, Latin America and Caribbean as well as in South East Asia [7–10]. Meanwhile, the afromontane areas characterized with unique biota [11], that had hitherto been known for being malaria free zones due to altitudinal effect, have seen increased malaria incidences with some areas experiencing a rise while others declining [12, 13]. Malaria cases have lately been observed to be on the rise in the afromontane ecotones within sub-Saharan Africa such as in the Rwenzori highlands of south western Uganda [14, 15]. Similar patterns have been experienced in the neighboring highlands of Butare (Rwanda) as well as in the Mount Kilimanjaro area (Tanzania) [16, 17]. These patterns in malaria have led to increased cost of malaria interventions [16, 18]. Such trends have been attributed to climate change that is creating ambient conditions within the highland altitudinal belts [18].

Malaria in Uganda has been endemic in the savannah areas of northern and eastern Uganda especially in Apac district, followed by Tororo district [19]. All these areas are within 1100 m altitude. However, highland areas especially Elgon region had earlier been reported to experience a surge in malaria cases despite continued intensified control and prevention interventions by both government, private sector and development partners [14, 16, 20]. These interventions have aimed at reducing malaria infections, reduce morbidity and prevent mortality attributable to malaria [21]. Control programs like the Uganda National Malaria Control Program (UNMCP) were developed based on the global Roll Back Malaria partnership, United Nations Millennium Development Goal and the 2000 Abuja Declaration [17, 21]. Implementation of the UNMCP plan is aimed at controlling malaria to reduce its burden on the human population in Uganda, ensure universal access to malaria prevention and treatment, and minimize mortality rate for children under 5 years of age. These strategies have involved integrated vector management, effective diagnosis and treatment, prevention of malaria in pregnancy, and attention to malaria epidemics [22]. Despite all these interventions, Uganda still ranks among the six countries that contribute more than half of the global malaria cases [1]. This is partly because of the climate which allows stable, year round malaria transmission with relatively little seasonal variability in most areas [17]. Within the country, malaria is highly endemic in up to 95% of the country’s area, where 90% of the population of 40 million live [22]. Despite inadequate information on the type and distribution of malaria parasites, the malaria species that are mainly reported in Uganda include *P. falciparum*, *P. vivax*, *P. malariae,* and *P. ovale* [23, 24]. *P. falciparum* is responsible for more than three quarters of the cases in Uganda [23]. It is estimated that other species account for < 5% of cases, with a few percent of infections due to mixed species [24].

Climate has been pointed out as a key risk factor for spatial-temporal patterns of malaria, especially in the highland areas [17]. Studies [19, 25] on malaria patterns in different mountainous areas have been undertaken but only a few [26, 27] have focused on the patterns of malaria within different altitudinal zones (ecotones). Yet ecotones are characterized with varying environmental conditions that can influence mosquito biology and malaria patterns [28, 29]. These studies have not documented patterns of malaria following intensified control and prevention interventions in mountainous areas such as Elgon region. This study thus analysed malaria patterns across altitudinal zones of Mount Elgon, one of the areas in Uganda where intensive malaria control and prevention programs have been implemented.

## Methods

### Study area

**Fig. 1:**
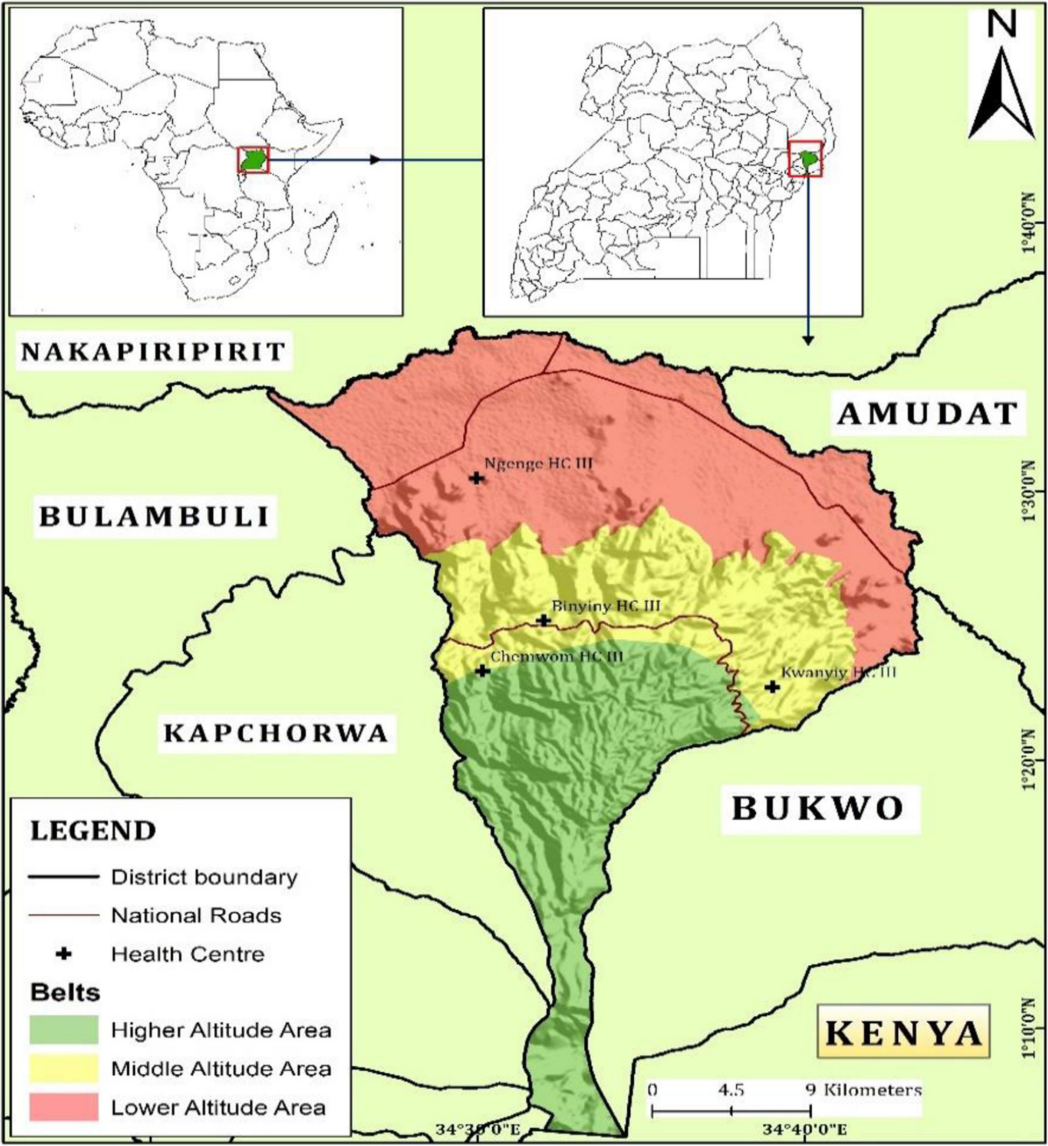
Location of Kween District (Source: Developed during this study)

The study was undertaken in the Mount Elgon highland region within Kween District located between 0125 N and 3431E (Fig. 1). Kween District borders the districts of Nakapiripirit to the north, Amudat to the northeast, Bukwo to the east, Kapchorwa to the west and Bulambuli to the northwest [28]. In the South, it boarders the Republic of Kenya and it is located on the northern slopes of Mount Elgon, at an average altitude of about 1900 m (6,200 Feet) above sea level [28]. It has administrative units ranging from Sub county, Parish and village [29]. The area is characterized by high and well-distributed rainfall (averaging 1200 mm/year) and consists of two seasons, a rainy season (March–September) and a dry season (October–April) [30]. It has cool temperatures which are on average 17 °C [31]. The human population of the district has been rising in the last three census conducted; 1991, 2002 and 2012 from 37,300, 67,200 to 103,300 respectively [32, 33]. Its population is majorly consisting of subsistence farmers cultivating a range of crops including: maize, beans, bananas, wheat, barley and cowpeas and also rear some livestock [28]. The district has health centers with levels: IV, III and II with numbers amounting to 1, 9 and 13 respectively [28]. These health centres are supported by a team of village health teams also known as heath service providers constituting Health Center I and are mainly responsible for mobilization of communities to access health services.

### Study design

This study employed a cross-sectional study design utilizing past malaria records from health center IIIs across the three altitude zones; higher (above 7150 ft), middle (between 4317 and 7150 ft) and lower altitude (below 4317 ft) of Mount Elgon [34]. Data on climate variables was obtained for the last 7 years (2011 to 2017). Data on confirmed malaria cases (using both microscopic and rapid diagnostic kits) from 2011 to 2017 was considered for this study and were computed to average number of true malaria cases per 1000 for each of the altitudinal zones. The rates of malaria cases were computed per month for each year. Climate data was obtained in retrospect for the 7 year period (2011 to 2017). Rainfall and temperature parameters (maximum and minimum) were the key climate parameters considered in this study as they play key roles in influencing breeding and survival of mosquitoes [35]. Analysis for the spatial temporal patterns was computed at parish level across the three altitudinal zones in the study area. Confounding factors like human population and vegetation were checked for their effect on the patterns of malaria incidences. Data on human population was obtained from the 2014 Uganda Bureau of Statistics records and computations were made for the values in different altitude zones using the human population growth rate. It was assumed that these human population values were a proxy to the actual population trends. Normalized Difference Vegetation Index (NDVI) was computed from high-resolution satellite images. Forecasts for malaria were made using ARIMA models for a period of 7 years (84 months) from the year 2017 [36]. Rates of malaria and time in terms of months were included in the model to understand the trends.

### Data collection

In this study, health centers from where data was collected were purposively selected basing on their capacity to confirm and report malaria cases, as well as the volume of their malaria records. Accordingly, the most suitable health centres that were used to collect data were the health center IIIs owing to their capacity to conduct malaria tests (both microscopic and Rapid Diagnostic Test kits). The cases selected for this study at least underwent through one of these tests but not both. These health centres were also fairly well distributed across the different altitude zones divided into higher (above 7150 ft), middle (between 4317 and 7150 ft) and lower altitudes (below 4317 ft) in the district. Data was then collected from four out of nine Health Center IIIs in the four sub-counties of Benet, Binyiny, Kwanyiy and Ngenge. Data on the number of malaria cases for the past 7 years was obtained from the Health Center IIIs records. Data collected included; malaria occurrence, parish of residence, tests as well as a range of socio-demographic characteristics (gender, age and location) of each patients were obtained for a period of 7 years.

Data for climate variables (temperature and rainfall) was obtained from the Uganda National Meteorological authority [32]. High resolution satellite images from 2011 to 2017 were downloaded from earth explorer (https://earthexplorer.usgs.gov/). Sentinel-2 images were downloaded for 2016 and 2017, Landsat 7 ETM+ (Enhanced Thematic Mapper Plus) images were downloaded for 2013, 2014 and 2015 while Landsat 5 TM (Thematic Mapper) images were downloaded for 2011 and 2012. The type of sensor used depended on the availability of clear images in a particular year.

### Data analysis

Malaria patterns were determined using descriptive statistics of means and standard deviations (SD). These were compared across different altitudinal zones; low, mid and high altitude. Mean malaria cases per month per 1000 cases were computed over the years (2011 to 2017) for each of the three altitude zones (Higher, Middle and Lower). Secondly, in order to depict the spatial-temporal variation of malaria cases, an Inverse Distance Weighted (IDW) interpolation regression [37] at a distance of 15 km was undertaken. The IDW is a deterministic regression procedure that estimates values at prediction points (V) using the following equation [38]:

$$ V=\frac{\sum_{i=1}^n{V}_1\left({d}_{i\hat{\mkern6mu} p}\right)}{\sum_{i=1}^n\left({d}_{i\hat{\mkern6mu} p}\right)} $$

Where d is the distance between prediction and measurement points, V_1_ is the measured parameter value, and p is a power parameter. The advantage of IDW is that it uses non-Euclidean “path distances” for d. These path distances are calculated using an algorithm that accounts for the malaria cases from one cell to the next [39]. Trend analysis was performed to determine the variation of the patterns of malaria [40]. The average monthly numbers of malaria cases per 1000 were calculated for the full time-series (January 2009–December 2015). These were plotted to show temporal patterns in malaria and climate variables. The time series of malaria incidence was decomposed using seasonal-trend decomposition based on locally weighted regression to show: the seasonal pattern, the temporal trend and the residual variability. The time series data, the seasonal component, the trend component and the remainder component are denoted by *Y*_*t*_, S_*t*_*, T*_*t*_*, R*_*t*_ respectively, for month *t* = 1 to N, and:

$$ {Y}_t={S}_t+{T}_t+{R}_t $$

The parameter setting “periodic” was used for the seasonal extraction, and all other parameters were by default. In the study, logarithmic transformations were used for the time series data [40].

Mann Kendal trend test [41] was used to detect the actual trends of the climate parameters and malaria. Relational analysis for malaria, temperature (maximum and minimum), human population, NDVI and rainfall was done using Kendall correlation. Model fitting was then performed to detect actual trends and relationships among variables. All analysis was done in R studio version 3.6.3 [42].

The Normalized Difference Vegetation Index was analyzed from the collected images by considering the red and near infra-red wavelengths bands in the respective images [43]. Prior to image analysis of Landsat 7 ETM+ images, a Landsat toolkit was used to remove scanline errors in all the images. NDVI was then computed by the following formula according to [44].

$$ \mathbf{NDVI}=\frac{\left(\boldsymbol{NIR}\hbox{-} \boldsymbol{Red}\right)}{\left(\boldsymbol{NIR}+\boldsymbol{Red}\right)} $$

## Results

### Trends in malaria across altitudinal zones

**Fig. 2:**
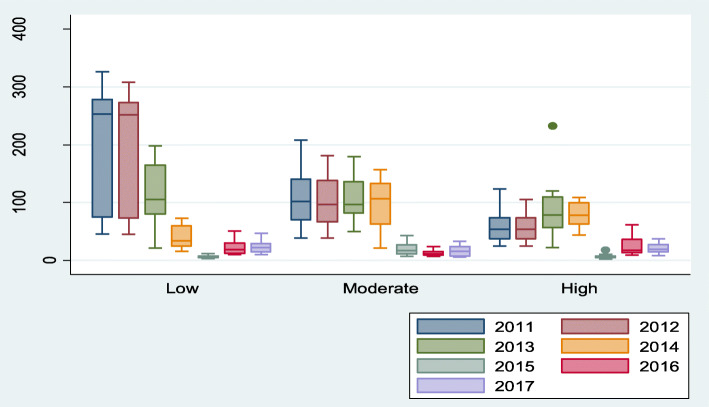
Malaria patterns across different altitudes of Kween District

**Fig. 3:**
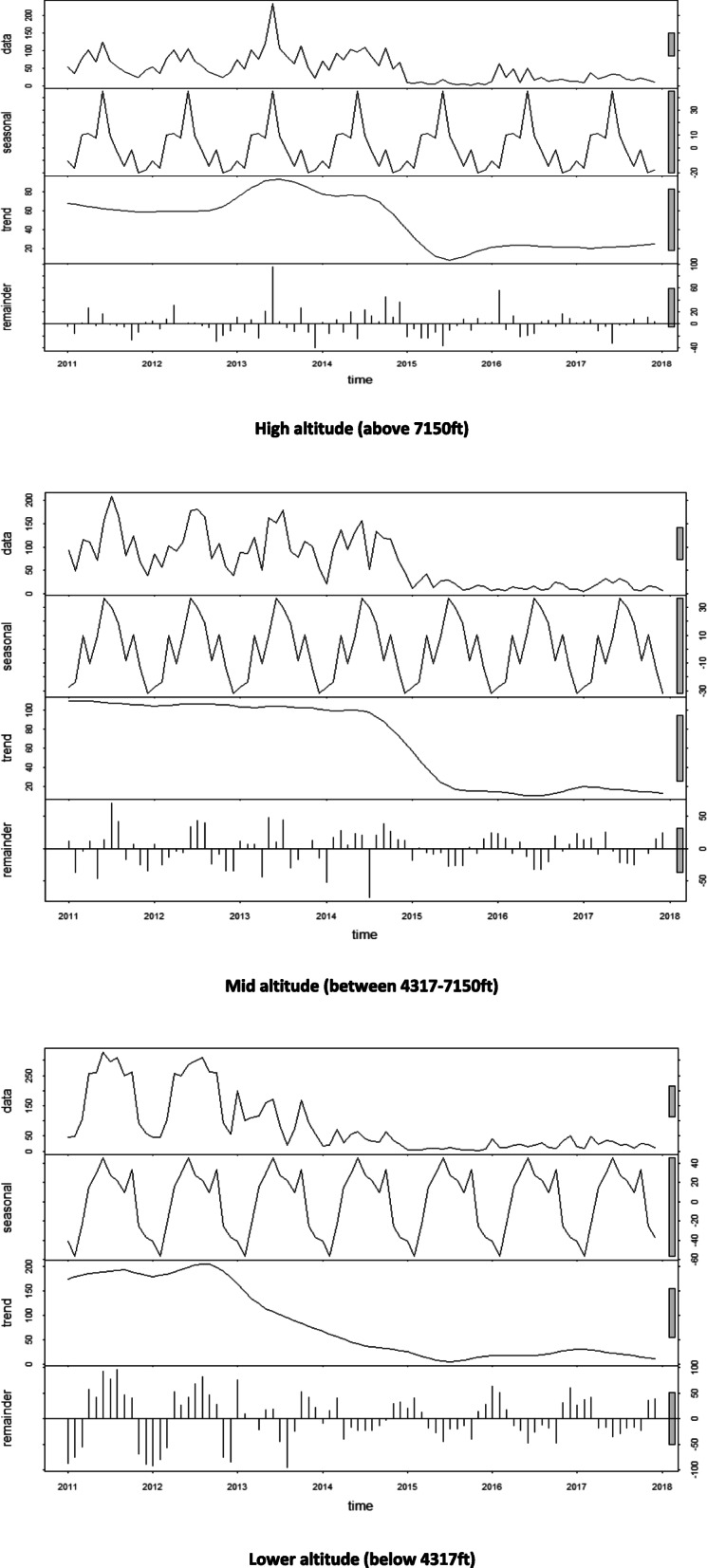
Trends of malaria cases (per 1000) from 2011 to 2017 in high, mid and lower altitudes of Kween District

Time series decomposition of malaria patterns revealed existence of seasonality of malaria across the years (2011–2017) in all the altitude zones (Fig. 3). The number of cases of malaria declined from 2011 to least number of cases towards 2017 (Fig. 3). There was statistical significant difference (*p* < 0.05) in the number malaria cases per 1000 individuals across the three altitude zones (lower, mid and higher altitude) in each of the years (2011–2017) except the years 2013 and 2017 (Fig. 2).

The cases of malaria per 1000 in high, mid and lower altitude areas were 49 (SD = 40), 67 (SD = 55) and 84 (SD = 96) respectively. Malaria cases revealed a normal curve-shaped trend over each year in the three areas (lower, middle and higher altitude areas) (Fig. 3). Also the months of June revealed highest numbers of malaria cases (94, SD = 73; 103, SD = 73 and 128, SD = 134 in high, mid and lower altitudes respectively) over the years (2011 to 2017). The months of January (41, SD = 29; 45, SD = 41 and 52, SD = 67 in high, mid and lower altitudes respectively) and December (28, SD = 23; 29, SD = 21 and 38, SD = 23 in high, mid and lower altitudes respectively) had the least number of malaria cases.

Results of the trend analysis through the Mann-Kendal trend test revealed a Sen’s slope of − 29.0 and − 10.9 (CI = 0.95) for malaria cases in the periods of March to September and October to February respectively in the higher altitude areas of Kween district. It also revealed a drastic decline of malaria cases over the 7 year period (from 2011 to 2017) with Sen’s value of − 21.5 (CI = 0.95). In the middle altitude areas, the Sen’s slope were − 44.8, − 56.0 and − 29 annually, March to September, and October to February respectively (CI = 0.95). In the lower altitude, the Sen’s values were − 87.8, − 120.7 and − 41.9 annually, March to September and October to February respectively (CI = 0.95).

### Spatial patterns of malaria cases across altitudinal zones

**Fig. 4:**
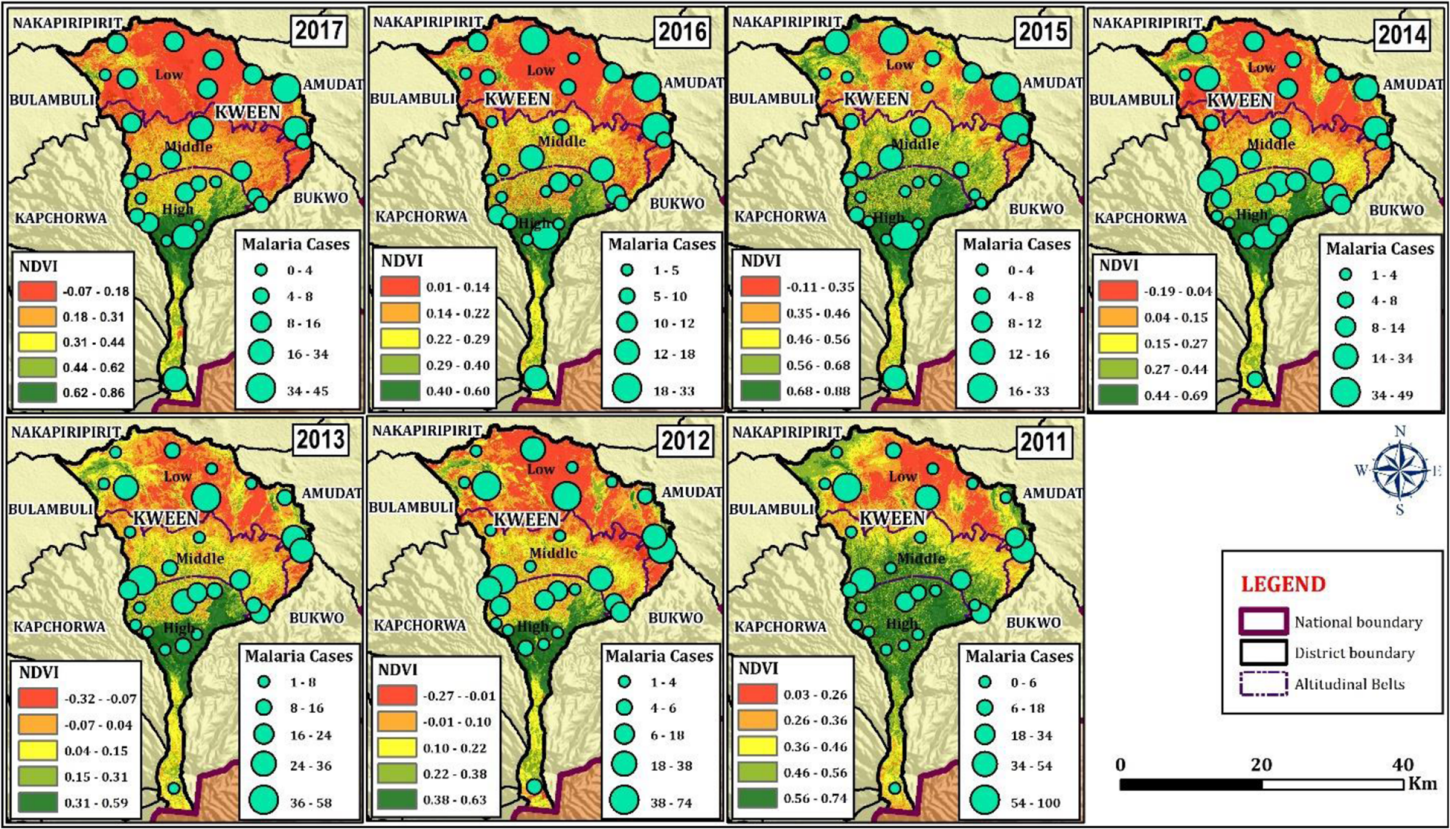
Spatial trends of malaria and NDVI across the different altitudes of Kween District from 2011 to 2017(Source: Images were developed during this study)

Spatial variation of malaria (Fig. 4) revealed higher number of cases of malaria in the lower altitude areas of Kween district. Higher and mid-altitude areas of the district had relatively lower number of malaria cases (49 ± 40 and 67 ± 55 respectively), while lower altitude areas had the highest (84 ± 96) number of malaria cases. The trends however declined from 2011 to 2017 in all the altitudinal zones (Fig. 4).

Regarding spatial variation of malaria cases with NDVI, there was an increase in malaria cases as the NDVI increases (Fig. 4).

### Biophysical and demographic factors interaction effect on malaria cases across altitudinal zones

**Fig. 5:**
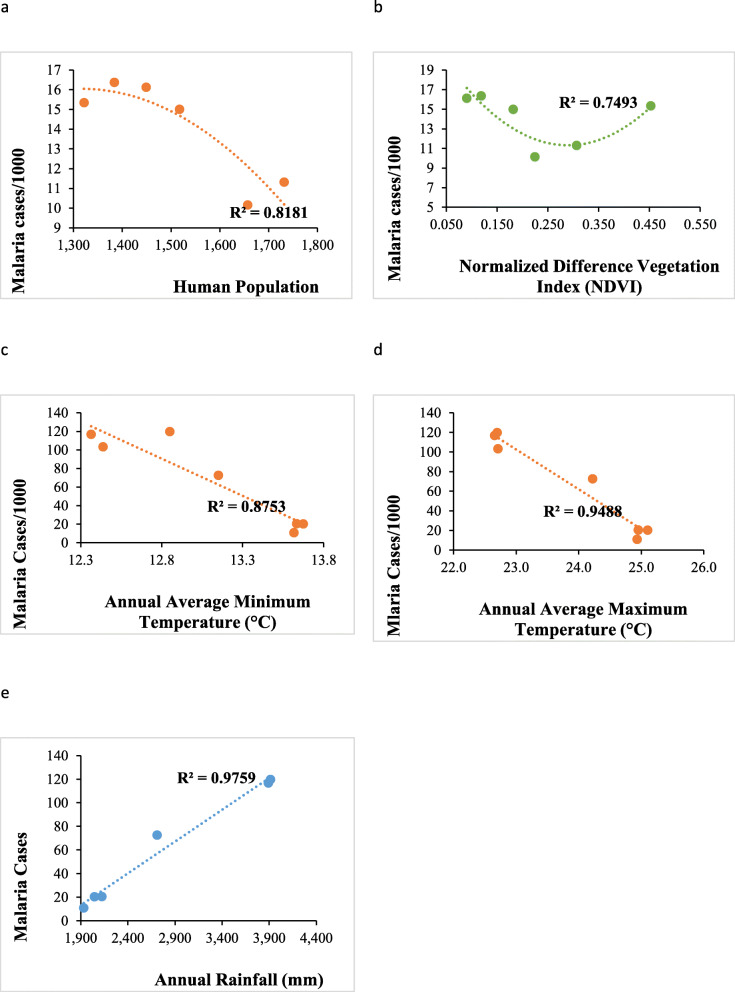
Fitted relationship between malaria patterns, vegetation cover and human population

We examined the relationship between biophysical factors; rainfall and vegetation and demographic factor-population and malaria cases across the altitudinal zones. Throughout the district, there was a good fit (*R*^*2*^ > 50%) of the model for the relationship between malaria and variables (human population, NDVI, rainfall, maximum and minimum temperature) values (Fig. 5). Malaria trends revealed a significantly positive correlation with the human population (*p* = 0.011) (Fig. 5a) and NDVI (*p* = 0.00069). Further, increase in vegetation cover in all the altitudinal zones caused a positive increase in malaria cases each of these zones. Meanwhile, increase in human population caused an increase in malaria cases (Fig. 5a).

**Fig. 6:**
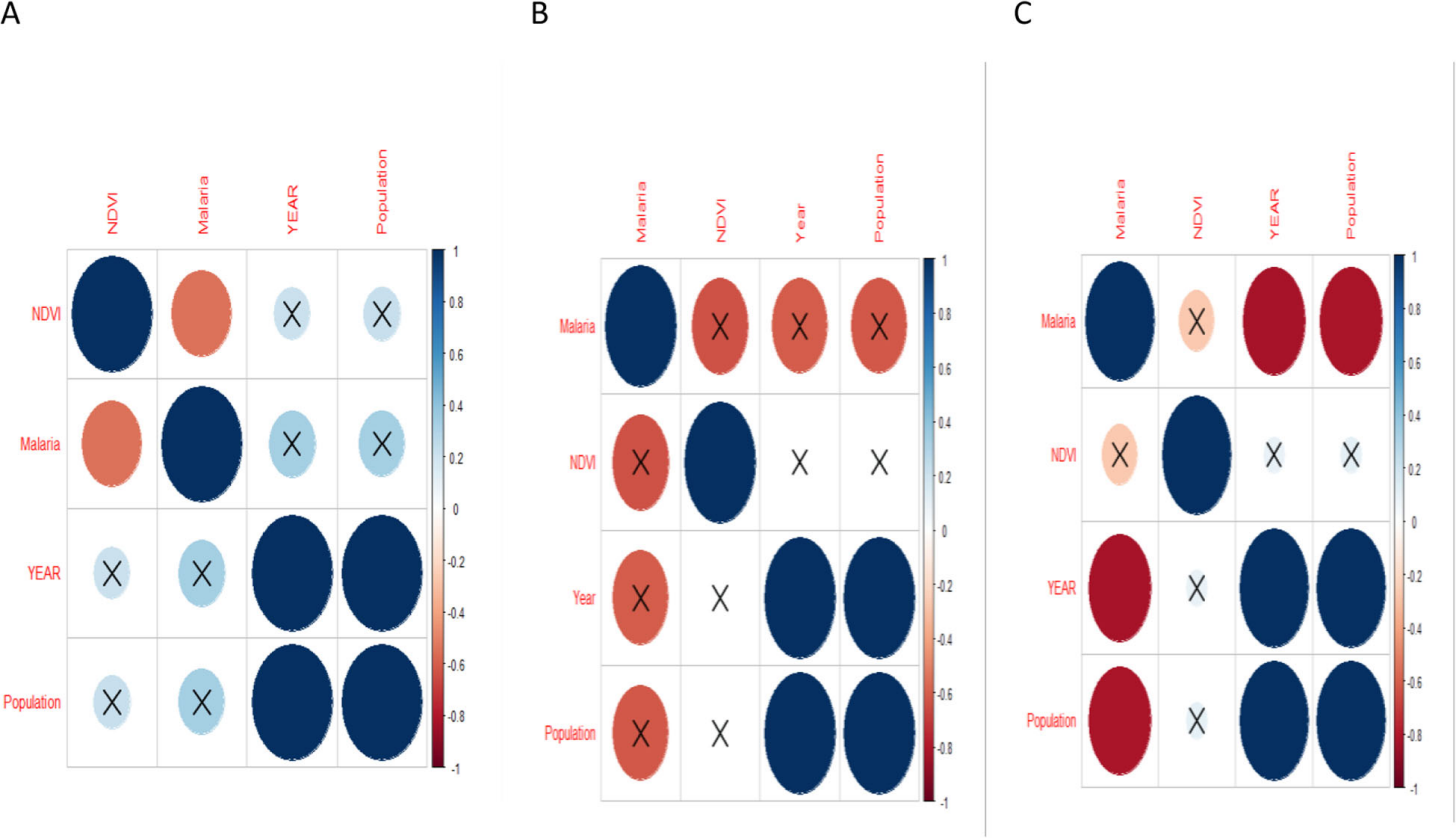
Relationship between malaria, vegetation cover and human population in the High (**a**), Mid (**b**) and Lower (**c**) altitudes of Kween District. (The size of the circles represent the relative size of the correlation value while crossed circles are values which were not significant at *p* < 0.05)

**Fig. 7:**
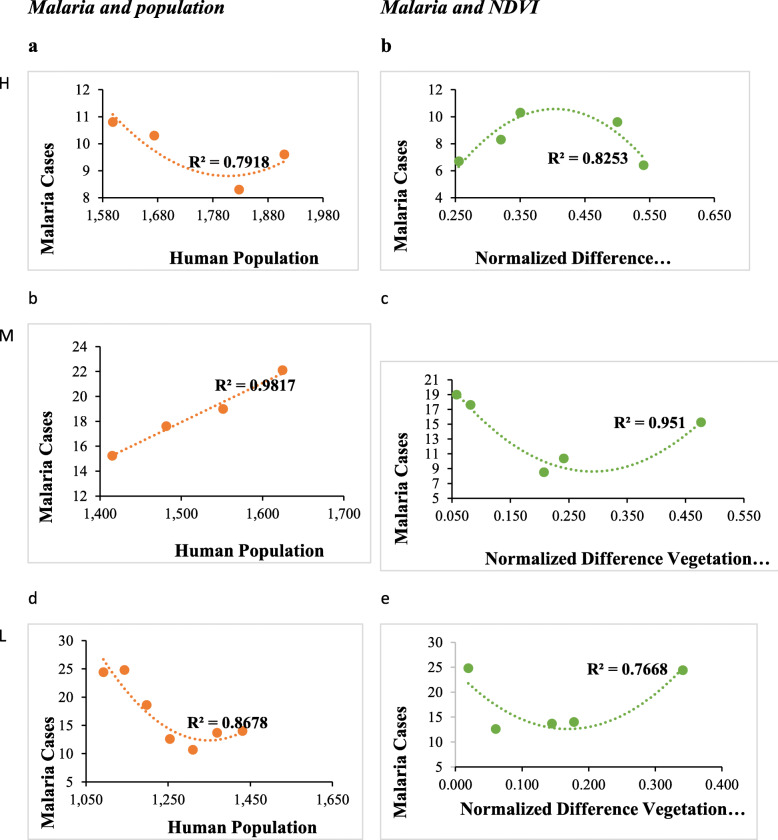
Fitted relationship between NDVI, human population and malaria in high (H), mid (M) and lower (L) altitudes

Altitudinally, the higher altitude areas had a positive correlation between human population and malaria. However, this correlation was not significant (Fig. 6a). The correlation between malaria cases and NDVI was significantly negative (at *p* < 0.05). In the mid altitude areas, malaria had a negative correlation with NDVI and human population (Fig. 6b). This negative correlation was however not significant. Lastly, in the low altitude areas, there was a negative correlation between malaria, human population and NDVI (Fig. 6b). The correlation between malaria and human population was significant. The R-squared values were higher (over 50%) reflecting a good fit of the model for this data (Fig. 7).

**Fig. 8:**
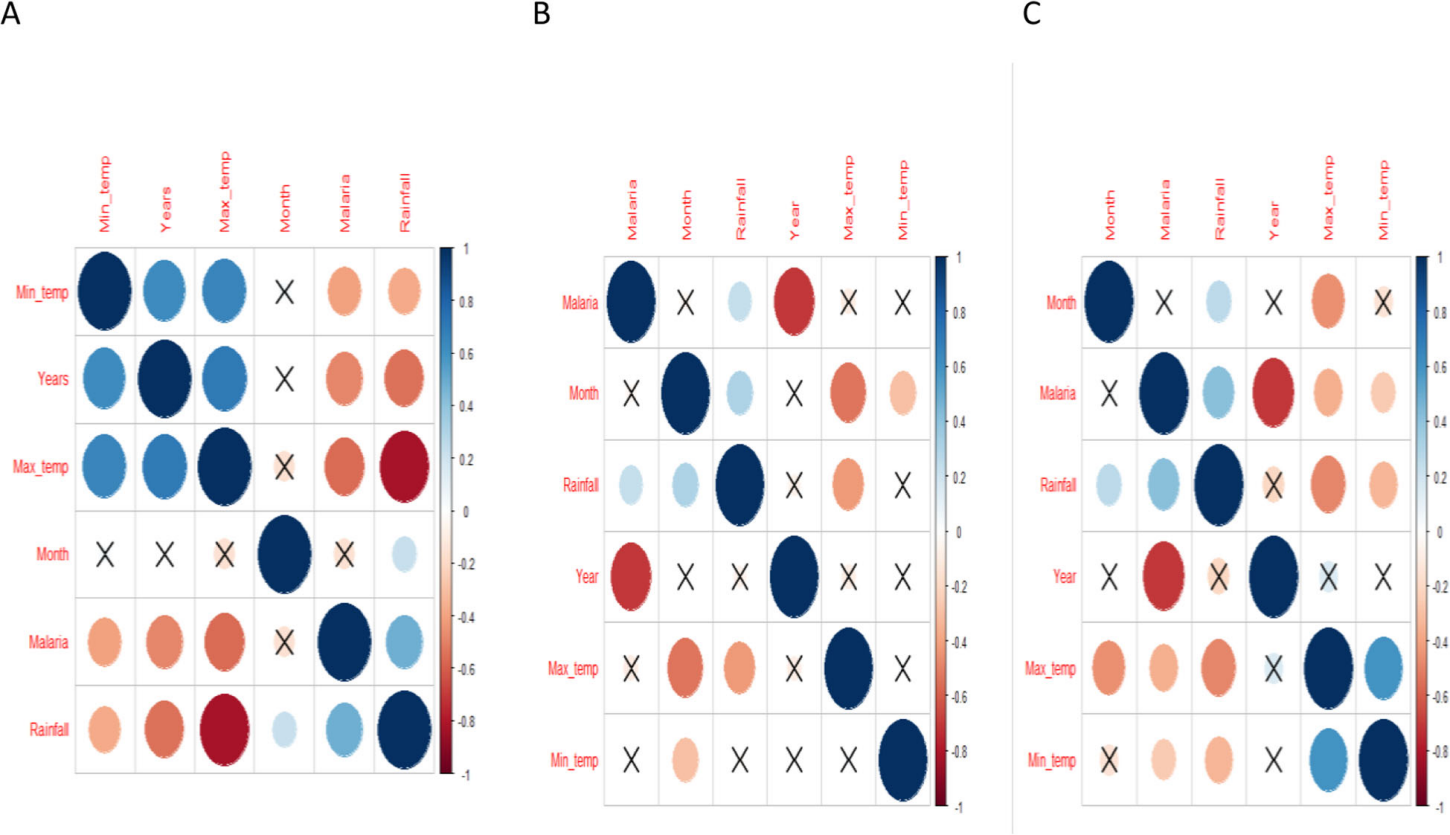
Relationship between malaria, minimum and maximum temperature and human in the High (**a**), Mid (**b**) and Lower (**c**) altitudes of Kween District. (The size of the circles represent the relative size of the correlation value while crossed circles are values which were not significant at *p* < 0.05)

Similarly, we analyzed effect of climate factors (rainfall, maximum and minimum temperature) on malaria patterns across the altitudinal zones. In the higher altitude areas, malaria had a significant negative correlation with maximum and minimum temperature. Maximum temperature had a higher negative value compared to minimum temperature (Table 1). Meanwhile, malaria cases recorded within these high altitude areas were significantly positively related with rainfall (Fig. 8a).

**Table 1:** Correlation between malaria, rainfall and temperature (maximum and minimum temperature in the altitudinal zones of Kween District

Altitude zone	Variables	*Corr*	*p-value*
Higher altitude	Malaria and Maximum Temperature	−0.42	**1.4e-08**
	Malaria and Minimum Temperature	−0.33	**7e-06**
	Malaria and Rainfall	0.32	**1.5e-05**
Mid altitude	Malaria and Maximum Temperature	−0.024	0.75
	Malaria and Minimum Temperature	−0.0095	0.9
	Malaria and Rainfall	0.17	**0.024**
Lower altitude	Malaria and Maximum Temperature	−0.17	**0.024**
	Malaria and Minimum Temperature	−0.087	0.24
	Malaria and Rainfall	0.21	**0.005**

In the mid altitude areas, malaria had a significantly positive correlation with rainfall (Fig. 8b; Table 1). However, there was very low and insignificant correlation between malaria, maximum and minimum temperature (Fig. 8b). Meanwhile, in the lower altitude areas, there was a significantly higher positive correlation between malaria and rainfall (Fig. 8c; Table 1). Meanwhile, malaria had a significantly negative correlation with minimum and maximum temperature (Fig. 8c). The correlation between malaria and maximum temperature was strongly negative compared to that of malaria and minimum temperature (Fig. 8c).

**Fig. 9:**
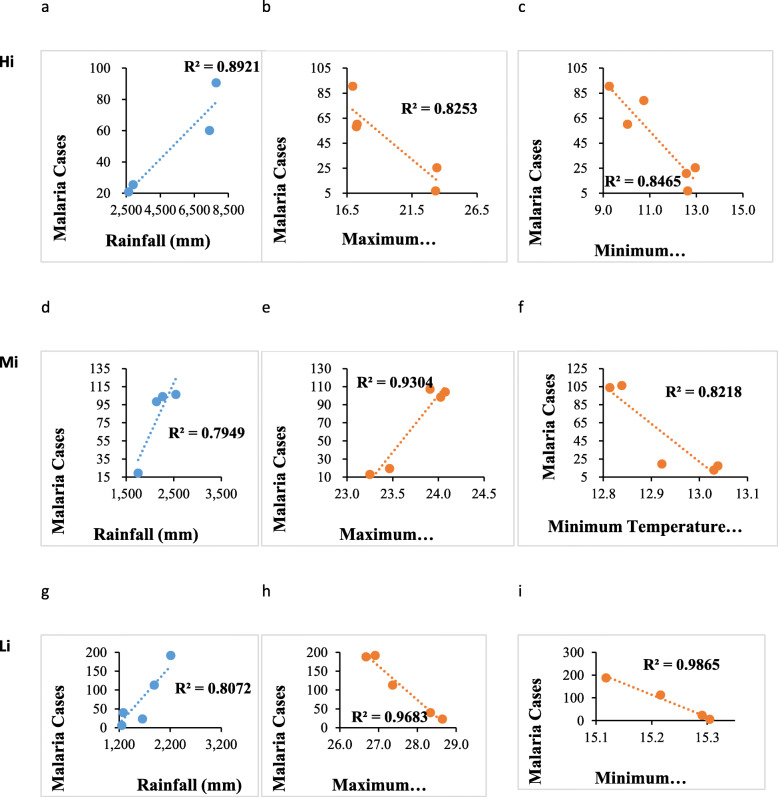
Fitted relationship between malaria and climate variable-rainfall, maximum and minimum temperature in high (Hi), mid (Mi) and lower (Li) altitudes

There was a good fit of the model reflecting effects of climate variables (rainfall, maximum and minimum temperature) (Fig. 9). Malaria trends in relation to the climate variables reflected similar trends as the correlation analysis except that of malaria and maximum temperature.

### Forecasting of malaria patterns across altitudinal zones

**Fig. 10:**
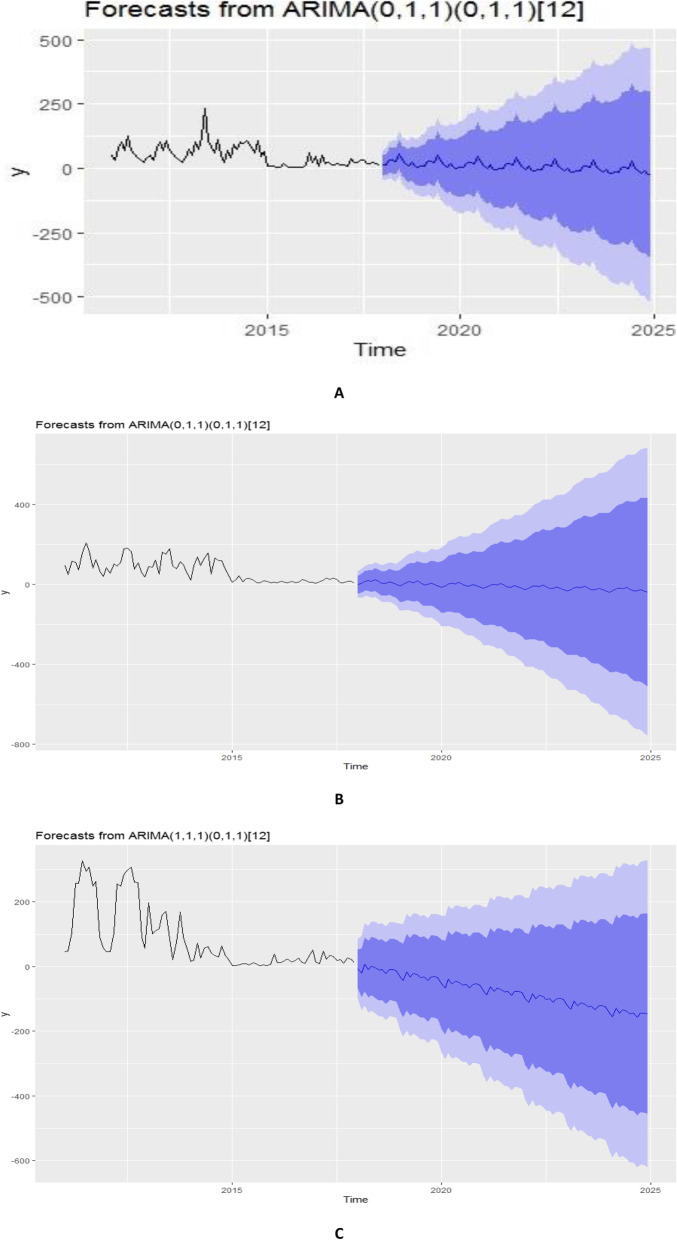
Forecasts of malaria patterns in the high (**a**), Mid (**b**) and Lower (**c**) altitude areas of Kween District

Forecasts of malaria for all the three altitudinal zones revealed malaria cases to continue to decrease for the following 7 (seven) years if the conditions were kept constant and/or intervention efforts are strengthened (Fig. 10). However, relaxation of the malaria control interventions would greatly allow for a surge in the cases of malaria (Fig. 10). Also, across the three zones, malaria appears to continue to be sustained in the high and mid altitude zones while the lower altitude zones would experience a decline in cases of malaria.

## Discussions

There was a declining number of malaria cases across all the altitudinal zones (high, mid and low altitudes) during the study period. This can be attributed to the intensified malaria control and prevention interventions within the study area, and also throughout the whole of Uganda. Intervention efforts by the Ministry of Health in malaria prevention and control through increasing access to health services including basic diagnostics, provision of insecticide-treated mosquito nets could have reduced malaria transmission within the study area. Similar declining trends had been pointed out in other studies conducted throughout the country between 2009 to 2014 [45]. Conversely, this pattern is contrasts the results in other studies undertaken earlier in highland areas of Kenya that showed malaria incidence to increase over time [46]. This could be because of the difference in the intensity of the control interventions and other environmental factors that influenced transmission dynamics of malaria. Although a surge in malaria cases was expected in the strongest El Niño years of 2015 and 2016, it was not detected in this study. One of the reasons could be due to the continuing efforts to prevent malaria transmission in Uganda. Recent studies have highlighted distribution of insecticide treated mosquito nets to significantly reduce malaria cases in Uganda [47]. However, this result could have been masked by the under reporting of the malaria cases.

Malaria patterns revealed a normal curve trend of malaria with the highest peak being in the middle (June–August) of each of the 7 years (Fig. 3). This corresponded to the trends in temperature and precipitation. However, the months of January and December had the least number of malaria cases. This can be linked to the low precipitation amounts during this period limiting availability of water for breeding of mosquitoes. This trend is similar to the results on studies undertaken in highland areas like Mount Kenya where malaria was prevalent during dry seasons [46, 48]. This trend can be linked to the availability of conditions favorable for growth and development of mosquitoes that transmit malaria parasites. Increase in temperature and availability of water sources favors mosquito breeding and its transmission of malaria parasites [49].

Spatially, the hotspot of malaria varied over the 7 year period dominating the lowland areas of the district (Fig. 4). The highland areas had lower number of malaria cases compared to the lowland areas. There was a significant negative correlation between malaria patterns in the lower belt and temperature. Also, there was a significantly positive correlation between malaria and rainfall within the lower belt. In the mid altitude areas, malaria had a significant positive correlation with rainfall. Meanwhile in the high altitude areas, malaria had a significantly negative correlation with maximum and minimum temperature. Also, malaria had a significant positive correlation with rainfall.

Although previous studies noted the critical role of increasing temperature in causing surge in malaria within sub-Saharan Africa, recent studies have shown that, temperature at times can significantly reduce the vectorial capacity of the mosquitoes [50, 51]. Ambient conditions of temperature enhance transmission by influencing vector and parasite life cycles [27]. However, increased or reduced temperature beyond optimal ranges can undermine the life cycle of mosquitoes limiting its transmission of malaria parasites [52]. Studies have highlighted the biological amplification nature of temperature on mosquitoes [53–55]. This study showed that the mean temperatures within the three altitudes varied. The difference in the contribution of maximum temperature to malaria cases between different altitudes can be attributed to the differences in prevailing temperatures in the three zones. The lower and mid altitude areas being relatively warmer and the district (Kween) having only one rainfall season was probably the main limiting factor in malaria vector development in the highland, mid and low altitude zones. Hence the onset of rainfall increased the media for vector growth and development. While rainfall creates the media, ambient temperatures favor the development and survival rates of both vectors and parasites. These conditions can be attributed to the trends of malaria in the three zones (high, mid and low) of Kween District. The highly seasonal rainfall within the study area could have limited the growth and development of mosquitoes.

The pronounced malaria cases in the lower altitude zones compared to the higher altitude zones can be linked to the environmental conditions favorable for mosquito growth and development. The alternating trends can be alluded to temperature and rainfall as the latter can either favor or discourage optimal growth and development of mosquitoes [56]. In the low altitude areas where malaria had a significant relationship with malaria, it has been noted that temperature can determine the length of the time the mosquitos explore food resources while transmitting malaria [57]. This could be the same case in low altitude areas. This study was limited by lack of data on the actual malaria and mosquito vectors. This would have complemented information on understanding of the life cycle of the parasites. Future studies ought to incorporate these aspects.

Regarding effects of vegetation cover and human population on malaria, malaria had a significant negative correlation with NDVI. Similarly, in the lower altitude, malaria had a significant negative correlation with human population and NDVI. This implies vegetation increase significantly influenced malaria cases in the high altitude areas. Increase in vegetation enhances the habitat range for mosquitoes. This result has been highlighted in some studies that note vegetation cover to influence dynamics of growth and development of mosquitoes and that of the vector [58]. Also, increase in human population over time in these areas could have caused a decline in vegetation cover that would facilitate transmission of malaria by mosquitoes. Over time, extension of health services also with increasing human population could have contributed to the declines in malaria with increasing human population. Although the result regarding these two aspects in this study reveal interesting results, it was limited by the inability to disintegrate data into shorter time ranges. This was due to the unreliability of the data. Therefore future studies ought to further explore vegetation and population dynamics at monthly level and their effects on malaria incidences. This will generate information on how human activities influence transmission and incidences of such infectious diseases.

Forecasts of malaria patterns revealed a continued decline of malaria cases given conditions remain constant. However, the number of malaria cases may significantly explode if temperature and rainfall increase. This implies that interventions at this point ought to be intensified. There is also a window of opportunity for eradication of malaria in the event that the existing control and prevention interventions are intensified. This thus calls for more studies to inform modification of the interventions.

One of the limitations of this study was the use of data from ministry departments in Uganda. There is therefore no proof of validity of this data as some of it was not complete. However, it gives a general picture of what can be done so as to curtail malaria infections within high altitude areas.

## Conclusions

Malaria patterns decreased over the study period in all the zones. Also, malaria belt was highly variable in in the altitudinal zones with the higher altitude areas becoming hotspots at some periods. Rainfall played a significant role in the distribution of malaria across the three zones of high, mid and lower altitude. This calls for strengthening of malaria control interventions irrespective of altitudinal ranges. The government of Uganda ought to design strategic malaria interventions to cater for different altistude zones. Stakeholders involved in malaria control and eradication efforts ought to design location specific interventions for malaria factoring out other factors like rainfall that had earlier received less attention in influencing malaria transmission. More large-scale studies should be undertaken in an attempt to understand how climate and other environmental factors influence similar variations of malaria (including different species of malaria) in different altitudinal zones. These studies should ensure validity of data by undertaking prospective studies within the population.

## Data Availability

The datasets used and analyzed during this study are available from corresponding author on reasonable request.

## Abbreviations

NDVI: Normalized Difference Vegetation Index
ARIMA: Autoregressive Integrated Moving Average
WHO: World Health Organization
UNMCP: Uganda National Malaria Control Program
ETM +: Enhanced Thematic Mapper Plus
TM: Thematic Mapper
SD: Standard Deviation
IDW: Inverse Distance Weighted
